# “Extreme Nephroptosis”: A Kidney in the Inguinal Hernia

**DOI:** 10.1155/2023/1439919

**Published:** 2023-08-10

**Authors:** Dmytro Shchukin, Vladyslav Demchenko, Andrii Arkatov, Roman Stetsyshyn, Gennadii Khareba, Vladyslav Bielov

**Affiliations:** ^1^Kharkiv National Medical University, 4 Nauky Avenue, Kharkiv 61022, Ukraine; ^2^V.I. Shapoval Regional Medical Clinical Center of Urology and Nephrology, 195 Heroiv Kharkova Avenue, Kharkiv 61037, Ukraine; ^3^Kharkiv Medical Academy of Postgraduate Education, 58 Amosova Street, Kharkiv 61176, Ukraine; ^4^“Snezhana” Medical Center, 45 Tobolska Street, Kharkiv 61045, Ukraine

## Abstract

We present an extremely rare case of renal ptosis from the normal orthotopic position into the cavity of inguinal hernia in a 93-year-old male patient. The following clinical case was accompanied by renal insufficiency, which was associated with the obstruction of the right ureter in the hernial sac and the stenosis of the left renal artery. The differential diagnosis between nephroptosis and dystopic kidney was based on MDCT scan images, which demonstrated the length of the right renal artery to be more than 20 cm. The patient underwent percutaneous nephrostomy through the right inguinal area and was successfully followed up for two years. We also analyzed six similar clinical cases described in the literature. This disease has, thus far, been observed exclusively in elderly men with long-standing and large inguinal hernias. The most frequent complications in these patients include ureteral strangulation in the area of the hernial gate and renal failure.

## 1. Introduction

Renal ptosis (floating kidney) is an uncommon urological disease that continues to cause controversies among clinicians, despite more than a century long history of research on this pathology. The disease has many unique peculiarities [1]. At the end of the 19^th^ and early 20^th^ centuries, nephropexy was used to be one of the most widespread surgeries, as floating kidneys were considered to be the most frequent cause of abdominal pains, as well as certain neurological and psychiatric diseases. This explains the largest number of surgical techniques that were proposed for the correction of this particular surgical disease.

Another important feature is the fact that the degree of nephroptosis often does not correlate with the symptoms, with some patients experiencing severe pain despite minor renal displacement, while others with an expressed floating kidney showing no symptoms at all. In the management of patients with nephroptosis, one more point must be taken into account; despite significant progress in the field of visual and laboratory diagnostics, the differential diagnosis of pain associated with nephroptosis remains challenging in certain cases. Therefore, predicting the outcomes of surgical intervention in patients with nephroptosis is extremely difficult, as symptoms may persist despite the fixation of the kidney in its normal position.

The pathophysiological aspects of nephroptosis include the possible development of hydronephrosis due to ureteral obstruction and significant elongation of the renal vessels, leading to ischemic disorders or venous congestion in the kidney.

The most severe degree of renal ptosis is characterized by the kidney displacement of over three vertebral lengths and its localization in the pelvic cavity. The literature describes singular clinical observations of “extreme nephroptosis” when the floating kidney is located in the cavity of the inguinal-scrotal hernia [2–7]. In general, the inclusion of a parenchymal organ into an inguinal hernia is very rare. Such cases include the involvement of an ectopic testicle, ovary, uterus, accessory ectopic kidney, or transplanted kidney into the hernial sac [8].

We present a rare clinical case of significant renal ptosis with the kidney located in the cavity of the inguinal-scrotal hernia, which caused significant ureteral obstruction and required drainage of the pyelocaliceal system by percutaneous nephrostomy passed through the scrotum.

This case report has been reported in line with the SCARE criteria [9].

## 2. Case Presentation

A 93-year-old patient was admitted by the ambulance to the Regional Urological Clinical Center with complaints of a decrease in the urine output to 200 ml per day during the last three days, fever up to 38°С, mild pain in the right half of the scrotum area, and the general condition deterioration. There were no delays prior to the patient's assessment and subsequent treatment. The anamnesis revealed, that two months ago, he had a permanent urethral Foley catheter installed due to chronic urinary retention caused by benign prostatic hyperplasia (BPH). His past medical history included ischemic heart disease (with a myocardial infarction seven years ago), hypertension, and urolithiasis. In addition, five years ago, he experienced an ischemic cerebral stroke resulting in persistent left-side paraparesis.

The patient's general condition was assessed as severe. The patient was confused. The physical examination revealed large bilateral inguinal-scrotal hernias that he had been suffering from for the past 20 years. The abdomen was soft on palpation, moderately painful in the right iliac and suprapubic areas. There was mild tenderness during the palpation of the right half of the scrotum.

The patient's general blood count revealed leukocytosis (12 х 10^9^/L) with a shift in the leucocyte formula towards young forms and moderate anemia (Hb: 104 g/L; erythrocytes: 3.6 х 10^12^ g/L). The levels of creatinine and blood urea reached 240 *µ*mol/L and 14.8 mmol/L, respectively.

**Figure 1:**
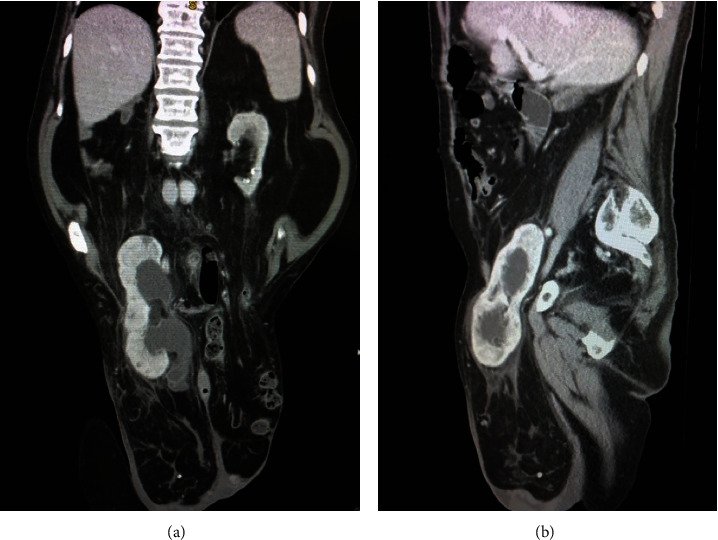
Multidetector-computed tomography (MDCT) demonstrates the location of the right kidney in the right-sided inguinal hernia. (a) Frontal reconstruction. (b) Sagittal reconstruction.

Ultrasonography revealed that the right kidney was located in the right half of the scrotum, with dimensions measuring 138 × 65 mm and a renal parenchyma thickness of 18–20 mm. There was significant extension of the pelvicalyceal system up to the level of the upper third of the ureter. The left kidney was situated in its typical position and measured 86 х 40 mm, with a parenchyma thickness not exceeding 10–12 mm. Calculi up to 12 mm were detected in the renal calyces. The renal collecting system of the left kidney was not dilated. There was suspicion on the pelvic dystopia of the right kidney, which was complicated by the displacement of the kidney with the hernial sac into the cavity of the right half of the scrotum. Considering the complexity of the given clinical presentation, the patient underwent a multidetector CT scan with contrast enhancement. The results of the CT scan confirmed the location of the right kidney in the right half of the scrotum (Figure 1).

**Figure 2:**
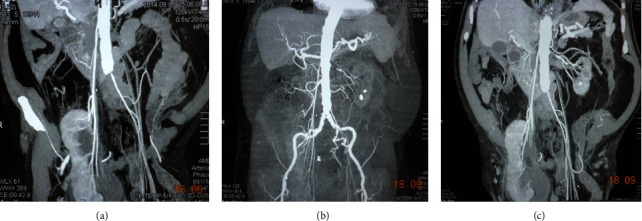
Multidetector-computed tomography. Frontal views present a severely elongated right renal artery and stenosis of the left renal artery (a–c).

However, the scrotal localization of the right kidney was a consequence of the nephroptosis rather than dystopia. This was objectively confirmed by the arterial phase of the investigation, which demonstrated that the ostium of the right renal artery was situated in the typical location (at the level L1-L2) and that the renal artery length had a length of over 20 cm (Figure 2).

Only the adipose tissue and the lower half of the right kidney were located within the hernial sac on the right side. The left-sided hernia contained loops of the small intestine and the omentum. The collecting system of the right kidney was dilated due to ureteral angulation. CT images demonstrated the small left kidney with significant stenosis of the left renal artery. Calyceal renal stones were also found in the left kidney without signs of obstruction.

**Figure 3:**
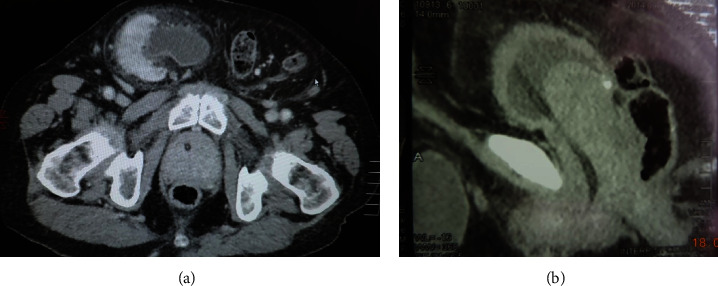
MDCT. Axial (a) and sagittal (b) views of an enlarged prostate gland and the right kidney located in the right half of the scrotum.

The CT scan also revealed an enlarged prostate measuring 90 × 65 × 60 mm with an intravesical component, as well as bladder stones (Figure 3).

**Figure 4:**
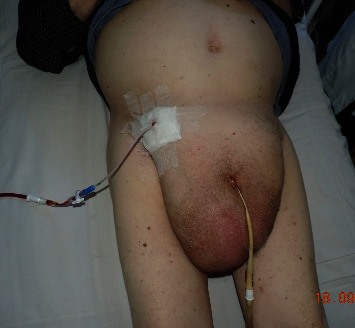
Percutaneous nephrostomy via the right inguinal area.

Obstruction of the right kidney due to ureteral strangulation in the hernia gate and stenosis of the left renal artery with the left kidney atrophy were the causes of oligoanuria in this patient. Considering the signs of acute right-sided pyelonephritis, signs of ureteral obstruction, serious overall condition, and the absence of intestinal loops in the hernial sac, it was decided to perform a minimally invasive treatment—percutaneous nephrostomy through the upper part of the right half of the scrotum (Figure 4). The nephrostomy was performed by the highest category urologist with over 20-year experience, without any difficulties or complications.

Over the next week, antibacterial and detoxication therapy showed favorable results. Diuresis through the percutaneous nephrostomy was approximately 2000–2500 ml/day and through the urethral catheter was approximately 200–300 ml/day. Given the patient's age, serious overall condition, and high risk of fatal complications during any surgical treatment, as well as the patient's will, it was decided to provide conservative treatment only. The patient was discharged with significant improvement. Over the next two years, he underwent periodic replacements of nephrostomy and urethral catheters performed by the same specialist as initial treatment.

## 3. Discussion

Inguinal hernia is one of the most common surgical diseases, which occur in about 3-4% of the people in the general population. This condition can lead to intestinal obstruction since the hernial sac typically contains the small intestine or omentum. While urological organs are less commonly involved in inguinal hernias, the bladder and ureter may also be affected. According to Oruç et al., 11.2% of hernias are associated with urological tumors and 23.5% are accompanied by complications [8]. Currently, only a few cases of “extreme nephroptosis” (the displacement of the kidney into the cavity of the inguinal hernia) have been reported [2–7].

We analyzed data from seven patients reported in the literature, including one of our own patients (Table 1). In all the cases, the patients were males. Their ages ranged from 62 to 93 years and averaged 79.8 ± 11.2 years. In six out of the seven observations, right-sided hernias occurred. Symptoms of a strangulated inguinal-scrotal hernia were found in only two patients. Interestingly, in most cases, no intestinal loops were found in the hernia cavity. The most common clinical sign was ureteral obstruction due to its strangulation in the hernia, which occurred in six patients. Examination revealed severe renal failure in three patients as follows: in two cases, it was associated with the obstruction of the ureter in a hernia and with a wrinkled contralateral kidney, while in one case, it was associated with the obstruction of the ureter in a hernia and with crush syndrome after a traumatic brain injury. Urolithiasis was present in two patients, while stenosis of the renal artery of the contralateral kidney was present in one.

It is worth noting that the treatment of patients with “extreme nephroptosis” in most cases is a difficult task. This is due to the massive size of the hernia, the poor general status, and the age of the patients. All seven men in our study had large or “giant” irreducible hernias, including the monstrous hernia described by Wendler et al. [3]. In addition, all patients had hernias for an extended period. Six out of seven patients had a poor general condition upon admission to the hospital.

Surgical hernia repair has been described in two reports, with nephropexy being performed in only one of those cases [4, 5]. In our patient, due to his advanced age and poor general condition, we opted for percutaneous nephrostomy. The nephrostomy tube was inserted into the scrotum through the right inguinal region under ultrasonographic guidance. Such treatment approach was chosen based on the CT image data, indicating the absence of intestinal loops in the hernial sac and was not associated with any complications.

In six patients, CT images showed the kidney displacement into the scrotum from an orthotopic position. This conclusion was based on the significant elongation of the renal vessels. In our patient, the length of the right renal artery was over 20 cm. At the same time, there were no signs of wrinkling of this kidney. In the report by Osmani et al., the displacement of a pelvic dystopic kidney into the hernial sac was demonstrated [5].

Most probably, the mechanism behind the “extreme displacement” of the kidney into the inguinal hernia cavity is associated with the presence of wide hernial orifices and weak fixation of the perinephric fatty tissue to the sheets of Gerota's fascia.

Long-standing hernias can lead to highly unusual life-threatening complications. It is advisable to provide patients with education materials regarding possible hernia complications. The similar cases should be managed in specialized centers since they require regular follow-ups and repeated procedures by qualified surgeons.

## 4. Informed Consent

The patient provided a written consent for the medical intervention, as well as his permission for publication of all the clinical details and any materials associated with this procedure.

## 5. Conclusion

“Extreme nephroptosis” or the displacement of the kidney from its normal orthotopic position into the inguinal-scrotal hernia cavity is a very uncommon phenomenon that requires further investigation. This disease has, thus far, been observed exclusively in elderly men with long-standing and large inguinal hernias. The most frequent complications in these patients include ureteral strangulation in the area of the hernial gate and renal failure.

## Figures and Tables

**Table 1:** Clinical features of patients with “extreme nephroptosis.”

	Perone et al. [2]	Wendler et al. [3]	Farrell et al. [4]	Osmani et al. [5]	Cassidy et al. [6]	Hsabo et al. [7]	Own cases
Sex	М	M	M	М	M	M	М
Side	Right	Right	Left	Right	Right	Right	Right
Strangulated inguinal hernia symptoms	+	−	−	−	−	−	+
Wrinkled contralateral kidney	+	−	−	−	−	−	+
Renal insufficiency	+	+	−	−	−	−	+
Ureteral obstruction	+	+	+	+	−	+	+
No intestinal loops in hernia	+	?	+	+	?	?	+
Severe general status	+	+	−	+	+	+	+
Surgical treatment	?	?	Hernia repair, orchiectomy, and nephropexy	Hernia repair	−	−	Percutaneous nephrostomy via the scrotum

## Data Availability

The data used to support the findings of this study are included within the paper.
